# Factors associated with all-cause mortality in endovascularly treated patients with chronic limb-threatening ischemia

**DOI:** 10.3389/fepid.2026.1702848

**Published:** 2026-01-22

**Authors:** Mária Rašiová, Veronika Pavlíková, Marek Hudák, Viktor Kožár, Lucia Dekanová

**Affiliations:** Faculty of Medicine, Department of Angiology, East Slovak Institute of Cardiovascular Diseases, Šafárik University, Košice, Slovakia

**Keywords:** amputation, creatinine, ischemia, mortality, reintervention

## Abstract

**Background:**

Despite advances in treatment, mortality in patients with chronic limb-threatening ischemia (CLTI) is high. The aim of our study was to evaluate 5-year all-cause mortality and factors associated with it in endovascularly treated (EVT) patients with foot ischemic ulcers.

**Methods:**

We reviewed all patients who had undergone EVT for lower extremity peripheral artery disease between January 2016 and December 2018. Adjustments in multivariate analyses were performed for age, hypertension, diabetes mellitus, sex, smoking, dyslipidemia, chronic obstructive pulmonary disease, malignancy, atrial fibrillation, heart failure with reduced ejection fraction, coronary artery disease, postprocedural ipsilateral amputation, ipsilateral reintervention, number of endovascularly treated regions, fibrinogen and creatinine.

**Results:**

Four hundred and fifty-one patients (155 women, 296 men) with a mean age of 70.4 ± 9.60 years were included in the analysis. The 5-year all-cause mortality was 60.5%. In multivariate analysis mortality risk was higher in women (HR 1.42; 95% CI 1.09–1.86; *p* = 0.010), and after EVT in two or more anatomical regions (HR 1.37; 95% CI 1.05–1.79; *p* = 0.022). The mortality risk was positively associated with creatinine (HR 1.003; 95% CI 1.002–1.004; *p* < 0.001), and fibrinogen (HR 1.19; 95% CI 1.11–1.29; *p* < 0.001). Ipsilateral reintervention (HR 0.67; 95%CI 0.47–0.94; *p* = 0.021) and ipsilateral amputation after EVT (HR 0.71; 95% CI 0.51–0.98; *p* = 0.037) were associated with lower all-cause mortality risk.

**Conclusions:**

Female sex, treatment in two or more anatomical regions, creatinine and fibrinogen were associated with higher 5-year mortality risk. Lower 5-year all-cause mortality risk was observed in patients with ipsilateral reintervention and ipsilateral amputation after EVT.

## Introduction

Lower extremity peripheral artery disease (PAD) is the third leading cause of cardiovascular morbidity and mortality after coronary artery disease and stroke (1). More than 200 million people have PAD worldwide; chronic limb-threatening ischemia (CLTI) affects up to 11% of this population (2, 3). CLTI, the most severe manifestation of PAD, is defined by ischemic foot pain at rest, ischemic ulceration or gangrene. The therapeutic goals of patients with CLTI are to prolong survival, alleviate pain, facilitate ulcer healing, and enhance limb function as well as quality of life. Treatment includes medical therapy to reduce cardiovascular risk, revascularization to improve limb perfusion, and local care to control infection and improve wound healing (2). Endovascular treatment (EVT) facilitates wound healing by improving perfusion. However, CLTI mortality remains among the highest in cardiovascular diseases, with almost 40% of patients dead within 3 years (4). To improve survival, it is important to identify factors affecting the mortality of these patients.

The primary aim of the study was to determine 5-year all-cause mortality in patients with endovascularly treated CLTI. The secondary aim was to assess the association between sociodemographic information, comorbidities, laboratory parameters, treatment, selected endovascular factors and 5-year all-cause mortality.

## Materials and methods

The present study took place at the East Slovak Institute of Cardiovascular Diseases, a vascular center in Slovakia. We conducted a review of data from *de novo* patients with CLTI who underwent primary EVT between January 2016 and December 2018. The study was performed with the approval of the Ethics Committee of the East Slovak Institute of Cardiovascular Diseases (Košice, Slovakia, specific ethical approvement number A2082024). Prior to EVT, general informed consent was obtained for the possibility of processing all patient data. Patients with acral ischemic leg ulcers located on the toes, heel, dorsum of the foot, and lateral aspect of the foot were enrolled in the study. Patients who underwent angiography and were not suitable for EVT were not included in the study. Sociodemographic data (age, sex, body mass index, smoking status), patient treatment (anticoagulation therapy, antihypertensive therapy, statin therapy, proton pump inhibitor therapy), comorbidities (arterial hypertension, diabetes mellitus, chronic obstructive pulmonary disease [COPD], atrial fibrillation, coronary artery disease, dyslipoproteinemia, malignancy, heart failure with reduced ejection fraction [HFrEF]), number of endovascularly treated anatomical regions, amputations, ipsilateral reintervention, number of patent crural arteries after EVT, and selected laboratory parameters [LDL-cholesterol, HDL-cholesterol, creatinine, fibrinogen, estimated glomerular filtration rate (eGFR)] were collected from electronic medical records. Non-smokers were defined as individuals with a lifetime history of smoking fewer than 100 cigarettes. The diagnosis of CLTI and the localization of hemodynamically significant arterial lesions were assessed by duplex ultrasonographic examination. Morphological details were assessed by digital subtraction angiography. Coronary artery disease was defined as a history of typical angina, myocardial infarction confirmed by coronary angiography or CT-coronary angiography, either in the past or during hospitalization. COPD was defined as a previously diagnosed condition or active treatment of COPD. Hypertension was identified by systolic blood pressure ≥140 mmHg, diastolic blood pressure ≥90 mmHg, or requiring medication for blood pressure control. The diagnosis of diabetes mellitus was based on documentation and/or treatment with antidiabetic medications. Dyslipoproteinemia was defined by treatment with lipid-lowering drugs and/or LDL-cholesterol levels exceeding the recommended target (<1.4 mmol/L) (1). HFrEF was defined as a left ventricular ejection fraction ≤ 40%, diagnosed either before or during hospitalization via echocardiography. Atrial fibrillation was confirmed either in pre-hospital documentation or by electrocardiographic evidence during hospitalization, without differentiation between paroxysmal, persistent, or permanent types. Malignancy was defined as any malignancy in the perihospitalisation period and/or malignancy in the past. Chronic kidney disease was defined by documented chronic kidney disease with eGFR <60 mL/min/1.73 m^2^ of at least 3 months. The number of patent crural arteries after EVT was determined from the angiographic images, endovascularly treated anatomical regions were divided into aortoiliac, femoropopliteal and crural/pedal regions. All patients were treated with dual antiplatelet therapy for at least 1 month after EVT. After EVT, the wound was managed by a surgeon. The recommended arterial monitoring interval performed at our center was every 4 weeks until the wound healed, performed by duplex ultrasonography. Reintervention was indicated for ulcers with delayed healing accompanied by recurrent occlusion or stenosis of the artery on duplex ultrasonography. Minor amputations were those that involve the toes or portion of the foot, while major amputations imply more proximal limb loss e.g., below knee amputation or above knee amputation. Survival data were obtained from the Health Care Surveillance Authority Registry. This register recorded the date of death without specifying the death cause. Follow-up was initiated at the time of index EVT, with reinterventions and amputations occurring during follow-up and death recorded thereafter.

Categorical variables were expressed as counts and percentages and compared using the Chi-square test. The normality of continuous variables was assessed using the Shapiro–Wilk test. Parametric data were presented as mean and standard deviation, nonparametric data were presented as median with the 25th and 75th percentiles (lower and upper quartiles). The Student's *t*-test (for parametric data) and the Mann–Whitney *U*-test (for nonparametric data) were used to compare continuous variables between groups. Outliers were kept as part of the data set. Univariate and multivariate Cox regression models were performed to estimate the hazard ratio (HR) and 95% confidence interval. Adjustments in multivariate analyses were performed for age, hypertension, diabetes mellitus, sex, smoking, dyslipidemia, COPD, malignancy, atrial fibrillation, HFrEF, coronary artery disease, fibrinogen, creatinine, ipsilateral reintervention, ipsilateral amputation post EVT and number of endovascularly treated anatomical regions. Cut-off values were determined using a decision tree approach (CRT method, custom value 1). Continuous variables that were associated with 5-year mortality were binary coded using cut-off values. Differences were regarded as statistically significant at two-tailed *p*-values *p* < 0.05. All statistical analyses were performed using IBM SPSS Statistics for Windows, version 21.0 (IBM Corp., Armonk, NY).

## Results

A total number of four hundred and fifty-one patients (155 women, 296 men) with a mean age of 70.4 ± 9.60 years were included in the study.

Endovascular treatment involved the aortoiliac region in 10.4% of patients, the femoropopliteal region in 56.1%, and the crural/pedal region in 70.3% of patients. Treatment was performed in one anatomical region in 61.0% of patients, and in two or three regions in 39.0%. Minor secondary amputation after EVT was performed in 19.5% of patients, and major secondary amputation in 7.1% of patients.

**Figure 1:**
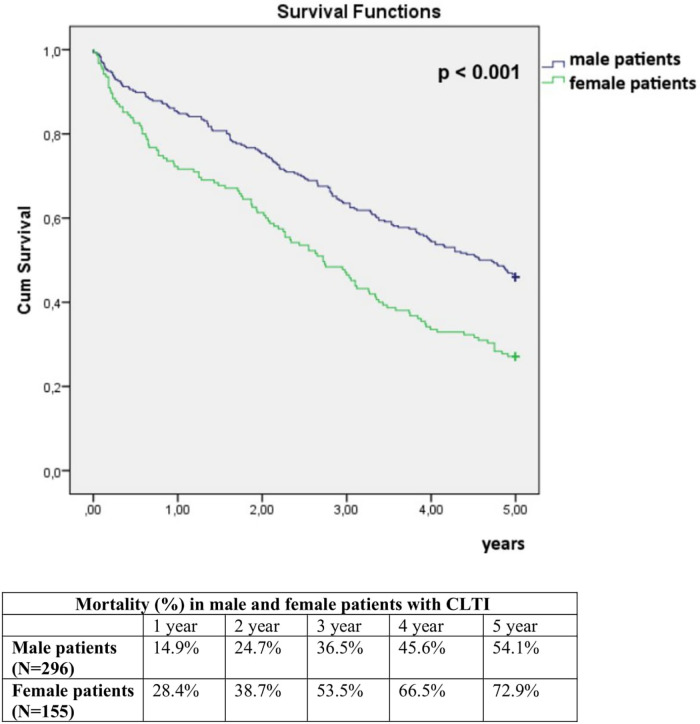
Comparison of 5-year mortality in male and female CLTI patients after endovascular treatment.

**Figure 2:**
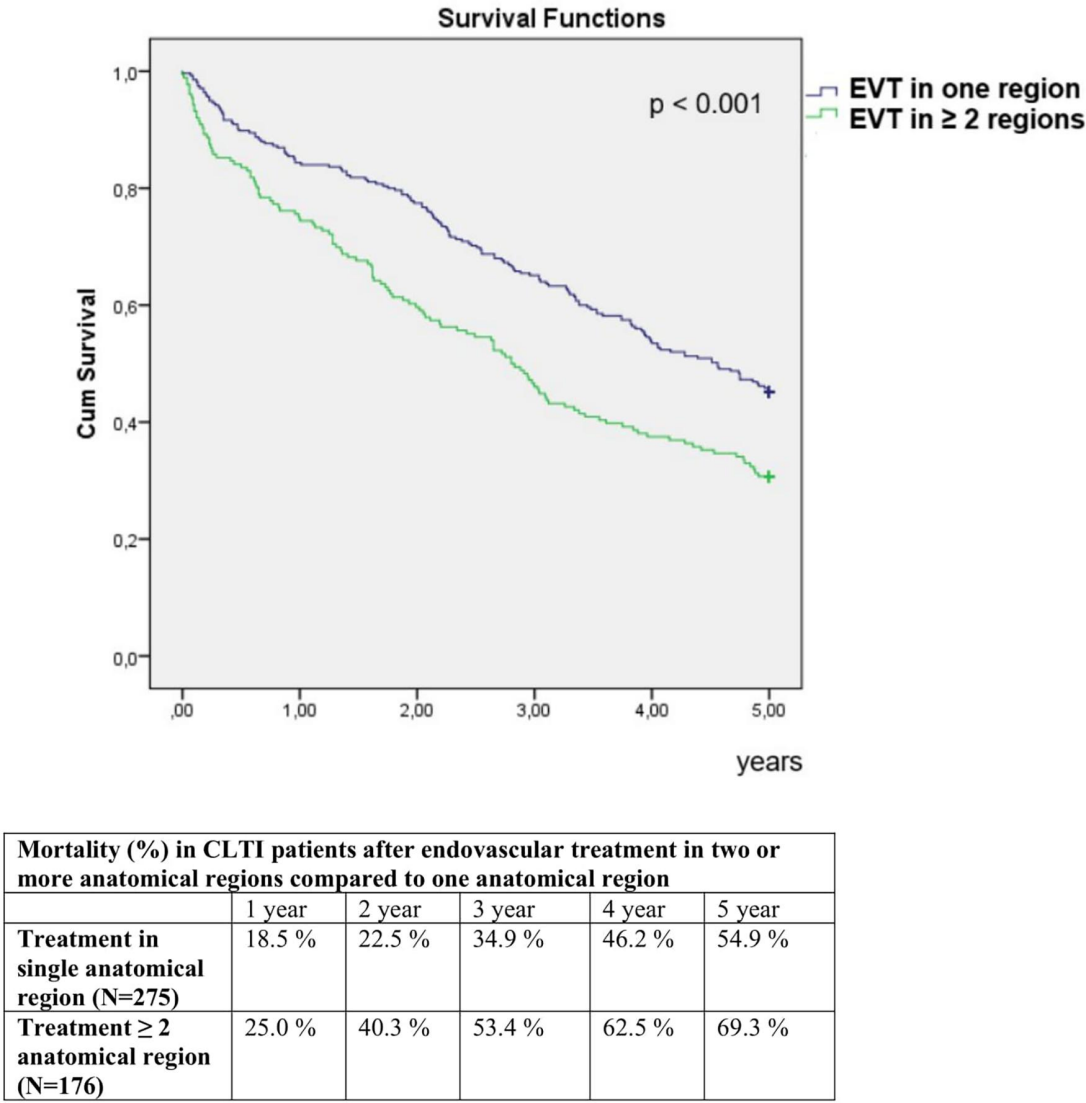
Comparison of 5-year mortality in CLTI patients depending on number of endovascularly treated anatomical regions.

**Figure 3:**
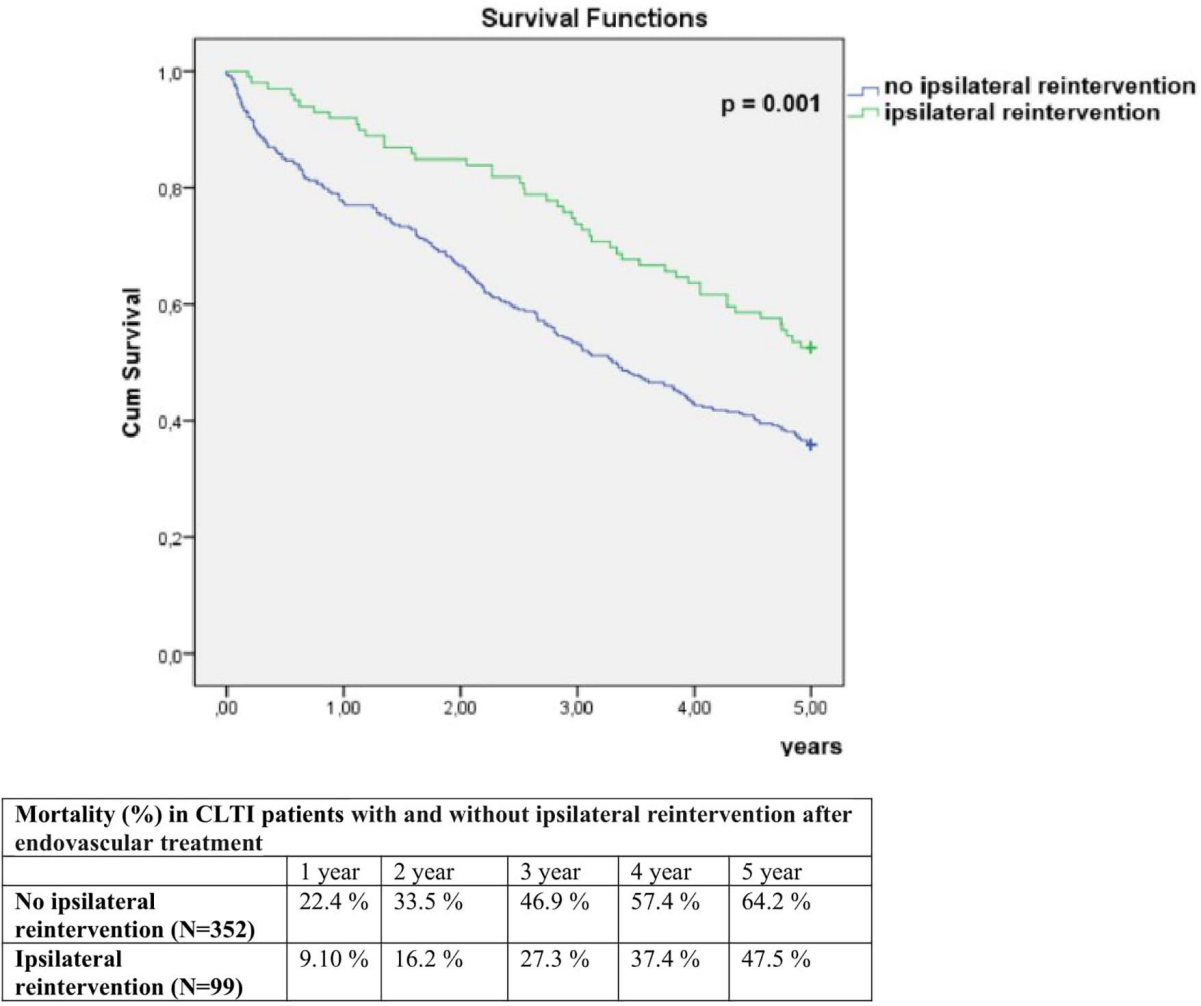
Comparison of 5-year mortality in CLTI patients with and without ipsilateral reintervention after endovascular treatment.

**Figure 4:**
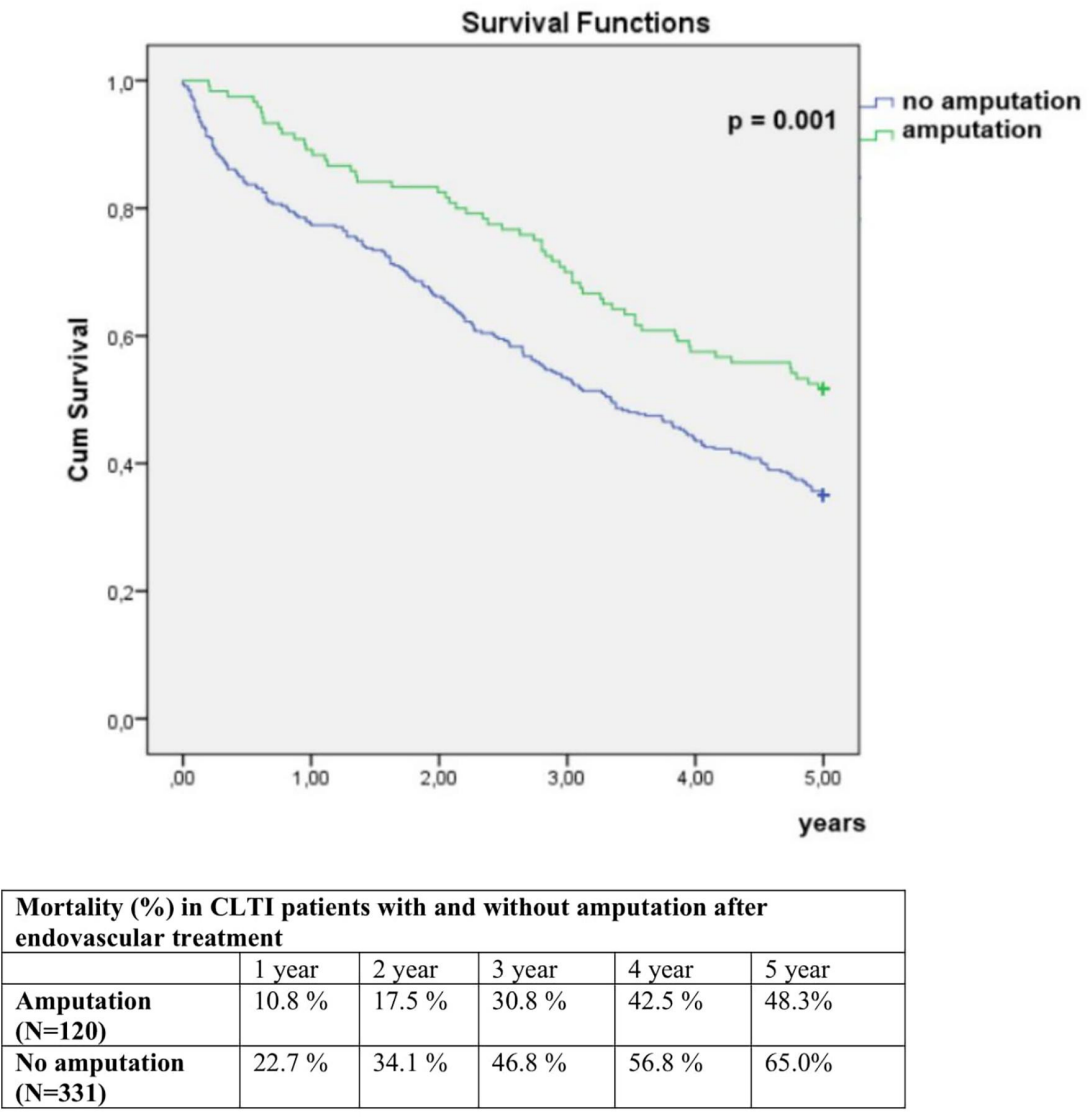
Comparison of 5-year mortality in CLTI patients with and without amputation after endovascular treatment.

The 5-year all-cause mortality was 60.5%. In univariate analysis, 5-year mortality was significantly higher in women compared to men (72.9% vs. 54.1%, *p* < 0.001), and in patients who underwent EVT in two or more anatomical regions compared to those treated in one region (69.3% vs. 54.9%, *p* = 0.002) (Figures 1, 2). Conversely, 5-year mortality was significantly lower in patients who underwent ipsilateral reintervention compared with those who did not (47.5% vs. 64.2%, *p* = 0.003), and in patients who underwent amputation compared with those who did not (48.3% vs. 65.0%, *p* = 0.001) (Figures 3, 4).

Mortality risk was positively associated with COPD (HR 1.61; 95%CI 1.07–2.41; *p* = 0.022), HFrEF (HR 1.66; 95%CI 1.16–2.37; *p* = 0.006), and chronic kidney disease (HR 1.69; 95%CI 1.28–2.23; *p* ˂ 0.001) in multivariate analysis.

In multivariate analysis, 5-year mortality risk was 1.42 times higher in women compared to men (HR 1.42; 95% CI 1.09–1.86; *p* = 0.010), and 1.37 times higher in patients who underwent EVT in two or more anatomical regions compared to one region (HR 1.37; 95% CI 1.05–1.79; *p* = 0.022). Conversely, 5-year mortality risk was 33.0% lower in patients with ipsilateral reintervention (HR 0.67; 95% CI 0.47–0.94; *p* = 0.021), and 29.0% lower in patients with amputation post-EVT (HR 0.71; 95% CI 0.51–0.98; *p* = 0.037). A trend toward lower mortality risk was observed in patients with major amputation (HR 0.77; 95% CI 0.58–1.03; *p* = 0.073).

**Figure 5:**
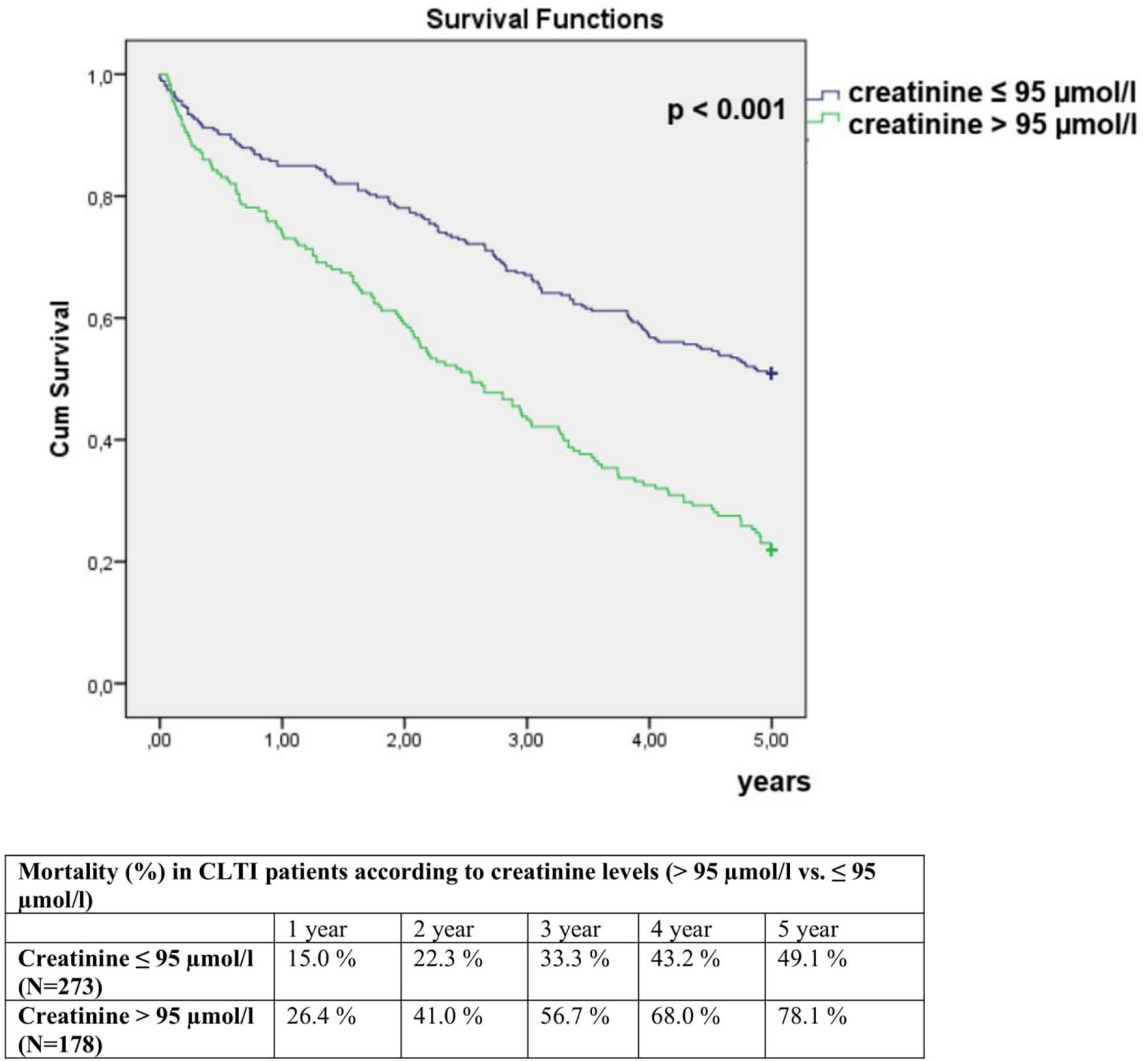
Comparison of 5-year mortality in CLTI patients with creatinine >95 µmol/L and ≤95 µmol/L.

Patients with creatinine >95 µmol/L had a 78.1% 5-year mortality compared to 49.1% mortality in patients with creatinine ≤95 µmol/L (*p* < 0.001) (Figure 5). The mortality risk in patients with creatinine >95 µmol/L was 1.70 times higher compared to patients with lower creatinine in multivariate analysis (HR 1.70; 95% CI 1.31–2.22; *p* < 0.001). A 1 µmol/L increase in creatinine was associated with a 0.30% increase in 5-year mortality risk in multivariate analysis (HR 1.003; 95% CI 1.002–1.004; *p* < 0.001).

**Figure 6:**
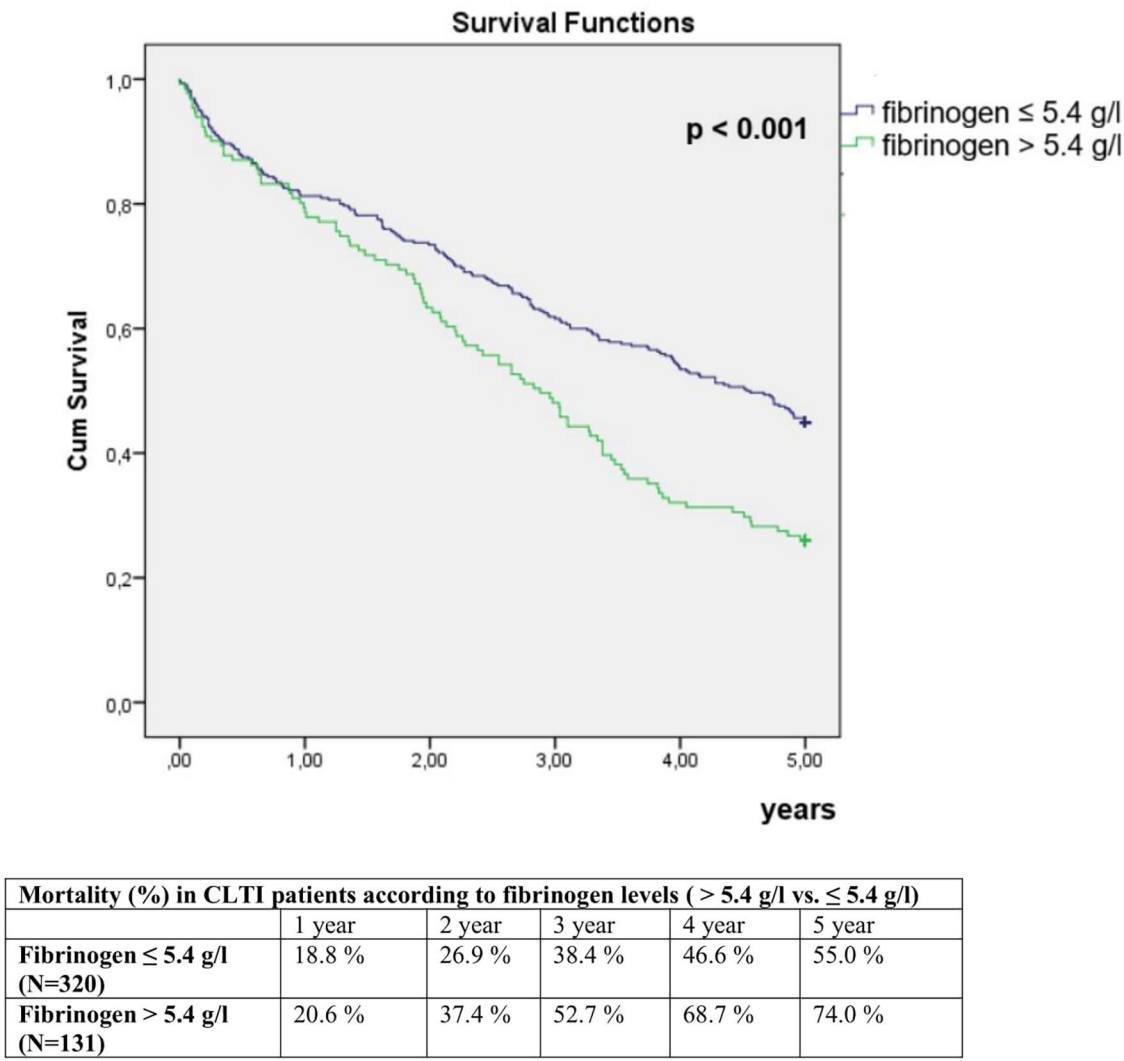
Comparison of 5-year mortality in CLTI patients with fibrinogen levels >5.4 g/L and ≤ 5.4 g/L.

Patients with fibrinogen >5.4 g/L had a 74.0% mortality compared to 55% mortality in patients with fibrinogen ≤5.4 g/L (*p* < 0.001) (Figure 6). Mortality risk in patients with fibrinogen >5.4 g/L was 1.61 times higher compared to patients with lower fibrinogen levels in multivariate analysis (HR 1.61; 95% CI 1.24–2.09; *p* < 0.001). A 1 g/L increase in fibrinogen was associated with a 19% increase in mortality risk in multivariate analysis (HR 1.19; 95% CI 1.11–1.29; *p* < 0.001).

No association was found between other demographic factors, comorbidities, pharmacotherapy, laboratory parameters, procedural factors and 5-year mortality in univariate and simultaneously multivariate analyses. The association between demographic factors, comorbidities, laboratory parameters, treatment and 5-year mortality of CLTI patients after EVT is presented in Table 1. Differences between between men and women with CLTI in demographic characteristics, comorbidities, laboratory parameters, and treatment modalities are shown in Table 2.

**Table 1:** Association between demographic factors, comorbidities, laboratory parameters, treatment, and 5-year mortality in CLTI patients after EVT.

Factors	All CLTI patients (*N* = 451) N (%)	5-year all-cause mortality (univariate analysis) HR (95%CI; *p*-value)	5-year all-cause mortality (multivariate analysis) *HR (95%CI; *p*-value)
Demographic factors			
Women	155 (34.4%)	1.71 (1.35–2.18; <0.001)	1.42 (1.09–1.86; 0.010)
Age (years) *¡*	70.4 ± 9.60	1.05 (1.03–1.06; <0.001)	1.04 (1.02–1.05; <0.001)
Smokers + exsmokers	151 (33.5%)	0.75 (0.63–0.88; <0.001)	0.94 (0.78–1.13; 0.516)
Body mass index (kg/m^2^)*¡*	28.3 ± 5.96	1.00 (0.98–1.02; 0.936)	0.99 (0.97–1.01; 0.294)
Comorbidities			
Coronary artery disease	256 (56.8%)	1.48 (1.16–1.90; 0.002)	1.17 (0.90–1.53; 0.239)
Chronic obstructive pulmonary disease	44 (9.8%)	1.49 (1.03–2.15; 0.035)	1.61 (1.07–2.41; 0.022)
Malignancy	34 (7.5%)	1.64 (1.09–2.46; 0.017)	1.22 (0.79–1.89; 0.373)
Atrial fibrillation	92 (20.4%)	1.91 (1.45–2.50; <0.001)	1.23 (0.91–1.66; 0.181)
HfrEF	52 (11.5%)	1.81 (1.30–2.52; <0.001)	1.66 (1.16–2.37; 0.006)
Chronic kidney disease with eGFR < 60 mL/min/1.73m^2^	177 (39.2%)	2.51 (1.30–2.51; <0.001)	1.69 (1.28–2.23; < 0.001)
Diabetes mellitus	324 (71.8%)	1.02 (0.78–1.34; 0.867)	1.20 (0.89–1.61; 0.237)
Hypertension	409 (90.7%)	1.64 (1.03–2.62; 0.037)	1.51 (0.91–2.51; 0.108)
Dyslipoproteinemia	388 (86.0%)	0.71 (0.51–0.97; 0.132)	0.70 (0.50–0.98; 0.239)
Ipsilateral minor amputation before EVT	142 (31.5%)	1.04 (0.81–1.34; 0.750)	1.10 (0.81–1.48; 0.550)
Contralateral amputation before EVT	74 (16.4%)	1.16 (0.86–1.58; 0.329)	1.11 (0.78–1.57; 0.573)
Pharmacotherapy after endovascular treatment			
Anticoagulation therapy	117 (25.9%)	1.70 (1.32–2.20; <0.001)	1.17 (0.81–1.68; 0.398)
Beta-blockers	276 (61.2%)	1.35 (1.05–1.74; 0.018)	1.01 (0.76–1.35; 0.947)
ACE-inhibitors/ARBs	320 (71.0%)	0.59 (0.46–0.76; < 0.001)	0.77 (0.57–1.04; 0.088)
Calcium channel blockers	197 (43.7%)	1.15 (0.91–1.4386; 0.240)	0.97 (0.75–1.27; 0.844)
Statins	235 (52.1%)	0.85 (0.67–1.08; 0.186)	0.99 (0.74–1.31; 0.933)
Proton pump inhibitors	96 (21.3%)	1.45 (1.10–1.92; 0.008)	1.30 (0.96–1.75; 0.088)
Preprocedural laboratory parameters			
Creatinine (µmol/L) °	86.9 (69.8; 115.0)	1.00 (1.002–1.003; <0.001)	1.003 (1.002–1.004;< 0.001)
Fibrinogen (g/L) *¡*	4.77 ± 1.63	1.14 (1.06–1.22; <0.001)	1.19 (1.11–1.29; <0.001)
HDL-cholesterol (mmol/L) *¡*	1.10 ± 0.32	0.81 (0.41–1.61; 0.550)	0.61 (0.28–1.32; 0.211)
LDL-cholesterol (mmol/L) °	2.69 (1.92; 3.44)	0.88 (0.71–1.10; 0.255)	0.90 (0.71–1.13; 0.359)
Procedural factors			
Two or three regions of treatment	176 (39.0%)	1.58 (1.25–2.01; <0.001)	1.37 (1.05–1.79; 0.022)
Two or three patent crural arteries after treatment	281 (62.3%)	0.79 (0.61–1.02; 0.070)	1.02 (0.77–1.35; 0.918)
EVT in the aortoiliac region	47 (10.4%)	1.04 (0.71–1.51; 0.860)	1.64 (1.06–2.53; 0.028)
EVT in the femoropopliteal region	253 (56.1%)	1.54 (1.21–1.97; 0.001)	1.20 (0.83–1.72; 0.337)
EVT in the crural/pedal arteries	317 (70.3%)	1.12 (0.86–1.46; 0.393)	0.81 (0.59–1.11; 0.190)
Ipsilateral amputation after EVT	120 (26.6%)	0.61 (0.45–0.81; 0.001)	0.71 (0.51–0.98; 0.037)
Ipsilateral reintervention after EVT	99 (22.0%)	0.58 (0.43–0.80; 0.001)	0.67 (0.47–0.94; 0.021)

*After adjustment including age, hypertension, diabetes mellitus, sex, smoking, dyslipidemia, chronic obstructive pulmonary disease, malignancy, atrial fibrillation, heart failure with reduced ejection fraction, coronary artery disease, postprocedural amputation, reintervention, number of EVT regions, fibrinogen and creatinine.

Categorical data expressed as counts with percentages; °non parametric data expressed as median (25th, 75th percentiles); *¡*parametric data expressed as mean and standard deviation;.

ACE, angiotensin-converting enzyme; ARBs, angiotensin II receptor blockers; CLTI: chronic limb-threatening ischemia; eGFR: estimated glomerular filtration rate; EVT: endovascular treatment; HFrEF: heart failure with reduced ejection fraction; HDL: high-density lipoprotein; HR: hazard ratio; LDL: low-density lipoprotein.

**Table 2:** Comparison of demographic factors, comorbidities, laboratory parameters, and treatment between men and women with CLTI.

Factors	All patients (*N* = 451) N (%)	Women (*N* = 155) N (%)	Men (*N* = 296) N (%)	*p*-value
Demographic factors				
Age (years) *¡*	70.4 ± 9.60	73.8 ± 10.2	68.6 ± 8.74	<0.001
Smokers + exsmokers	151 (33.5%)	29 (18.7%)	126 (42.6)	<0.001
Body mass index (kg/m^2^)*¡*	28.3 ± 5.96	27.9 ± 6.96	28.5 ± 5.36	0.290
Comorbidities				
Coronary artery disease	256 (56.8%)	90 (58.1%)	166 (56.1%)	0.686
Chronic obstructive pulmonary disease	44 (9.8%)	12 (7.7%)	32 (10.8%)	0.297
Malignancy	34 (7.5%)	12 (7.7%)	22 (7.4%)	0.906
Atrial fibrillation	92 (20.4%)	34 (21.9%)	58 (19.6%)	0.558
HfrEF	52 (11.5%)	16 (10.3%)	36 (12.2%)	0.561
Chronic kidney disease with eGFR < 60 mL/min/1.73m^2^	177 (39.2%)	80 (51.6%)	97 (32.8%)	<0.001
Diabetes mellitus	324 (71.8%)	106 (68.4%)	218 (73.6%)	0.238
Hypertension	409 (90.7%)	146 (94.2%	263 (88.9%)	0.064
Dyslipoproteinemia	388 (86.0%)	134 (86.5%)	254 (85.8%)	0.852
Ipsilateral minor amputation before EVT	142 (31.5%)	33 (21.3%)	109 (36.8%)	0.001
Contralateral amputation before EVT	74 (16.4%)	16 (10.3%)	58 (19.6%)	0.012
Pharmacotherapy after endovascular treatment				
Anticoagulation therapy	117 (25.9%)	40 (25.8%)	77 (26.0%)	0.993
Beta-blockers	276 (61.2%)	104 (67.1%)	172 (58.1%)	0.051
ACE-inhibitors/ARBs	320 (71.0%)	103 (66.5%)	217 (73.3%)	0.153
Calcium channel blockers	197 (43.7%)	76 (49.0%)	121 (40.9%)	0.086
Statins	235 (52.1%)	84 (54.2%)	151 (51.0%)	0.521
Proton pump inhibitors	96 (21.3%)	38 (24.5%)	58 (19.6%)	0.225
Preprocedural laboratory parameters				
Creatinine (µmol/L) °	86.9 (69.8; 115.0)	84.6 (63.9; 112.5)	87.0 (72.1; 117.5)	0.781
Fibrinogen (g/L) *¡*	4.77 ± 1.63	4.73 ± 1.74	4.80 ± 1.58	0.703
HDL-cholesterol (mmol/L) *¡*	1.10 ± 0.32	1.20 ± 0.32	1.04 ± 0.31	0.050
LDL-cholesterol (mmol/L) °	2.69 (1.92; 3.44)	3.04 (2.07; 3.76)	2.47 (1.87; 3.15)	0.028
Procedural factors				
Two or three regions of treatment	176 (39.0%)	67 (43.2%)	109 (36.8%)	0.186
Two or three patent crural arteries after treatment	281 (62.3%)	83 (54.5%)	198 (66.9%)	0.007
EVT in the aortoiliac region	47 (10.4%)	17 (11.0%)	30 (10.1%)	0.783
EVT in the femoropopliteal region	253 (56.1%)	105 (67.7%)	148 (50.0%)	<0.001
EVT in the crural/pedal arteries	317 (70.3%)	97 (62.6%)	220 (74.3%)	0.010
Ipsilateral amputation after EVT	120 (26.6%)	34 (21.9%)	86 (29.1%)	0.104
Ipsilateral reintervention after EVT	99 (22.0%)	34 (21.9%)	65 (22.0%)	0.995

Categorical data expressed as counts with percentages; °non parametric data expressed as median (25th, 75th percentiles); *¡*parametric data expressed as mean and standard deviation; ACE: angiotensin-converting enzyme; ARBs, angiotensin II receptor blockers; CLTI, chronic limb-threatening ischemia; eGFR, estimated glomerular filtration rate; EVT, endovascular treatment; HFrEF, heart failure with reduced ejection fraction; HDL, high-density lipoprotein; HR, hazard ratio; LDL, low-density lipoprotein.

## Discussion

Our study analyzed factors associated with 5-year all-cause mortality in endovascularly treated CLTI patients. Higher 5-year mortality risk was found in women, after EVT in two or more anatomical regions, and conversely, lower mortality risk was recorded in patients who underwent ipsilateral reintervention and ipsilateral amputation after EVT. Creatinine and fibrinogen were positively associated with 5-year mortality risk.

Despite advances in endovascular and surgical treatment and the increasing number of new drugs to treat risk factors such as hypertension, hyperlipidemia, and diabetes, CLTI remains an extremely morbid disease with a similar mortality rate over the past 20 years (4). During 5-year follow-up, the overall mortality rate in our study was 60.5%, which is consistent with the literature. The overall mortality rate in patients with CLTI is higher than for most malignancies; the 5-year combined mortality rate for all reported cancers was 31.0% in the study conducted by Armstrong et al. (5). After initial diagnosis of CLTI, the risk of mortality was approximately 20%–25% at 1 year and approximately 60% at 5 years (6, 7). After surgical or endovascular revascularization in non-elective patients, a 5-year mortality rate of 64.3% had been documented (8, 9).

Mortality reflects the polymorbidity of the patients and generalized atherosclerosis. In our study, 39.2% of patients had chronic kidney disease, 71.8% of patients had diabetes mellitus, 20.4% of patients had atrial fibrillation, 11.5% of patients had HfrEF, and 56.8% of patients were being treated for coronary artery disease.

In contrast to the high mortality observed in patients with CLTI, patients with claudication exhibit lower 5-year mortality risk, which was reported as 25.3% in the study conducted by Pavlikova et al. (10).

Five-year mortality risk was higher among women than among men. Women accounted for 34.4% of the study population and were older than men (73.8 ± 10.2 vs. 68.6 ± 8.74 years).

Women were more likely to be treated for chronic kidney disease (51.6% vs. 32.8%), had a lower prevalence of ipsilateral minor amputation before EVT (21.3% vs. 36.8%), and had lower crural artery patency after EVT, with two or three patent crural arteries observed in 54.5% of women compared with 66.9% of men.

Although the incidence and prevalence of CLTI are higher in men, female sex is associated with delayed diagnosis, a greater influence of nontraditional risk factors, atypical clinical presentation, and lower prescription rates of cardioprotective medications (11–15). Furthermore, women generally have smaller arterial diameters and appear to be at increased risk of stent thrombosis, as well as bleeding and wound-related complications (16).

According to the study conducted by Makowski et al. and Hata et al. women with CLTI treated with surgery or EVT had lower mortality compared to men (11, 12). On the other hand, the meta-analysis published by Faraga et al. documented a 1.17 times higher mortality risk in women compared to men after EVT or surgery (17).

Interestingly, the study published by Skoog et al., which followed 30-day mortality over three time periods (1994–1999; 2000–2006; and 2007–2013), confirmed an increasing mortality rate in CLTI patients, especially in women (30-day mortality in 2007–2013 was 5.4% in women and 4.9% in men) (18).

As there is diverse data in the literature that examines sex as a biological variable in CLTI, prospective studies with a larger inclusion of women are nedeed to understand possible sex-specific mechanisms in PAD (19, 20).

The most common cause of death in patients with CLTI in the acute stage is sepsis, followed by death from cardiovascular causes in the later period (21). Patients who underwent EVT in two or more anatomical regions were likely to have more diffuse atherosclerotic involvement in other arterial territories, which may have contributed to their higher mortality. Interestingly, higher mortality was documented in patients without ipsilateral reintervention. There are opinions that revascularization improves wound healing but does not affect patient mortality (21). Ipsilateral reinterventions were probably performed in patients who cooperated with follow-up after EVT, and were under closer medical supervision which may have positively affected their survival.

However, the observed improvement in survival among patients with CLTI undergoing amputation or reintervention should be interpreted with caution. Both amputation and reintervention represent time-dependent interventions, and the requirement to survive until the procedure introduces a risk of immortal time bias, potentially favoring the intervention groups. Furthermore, survivorship bias may be present, as patients who were clinically stable enough to undergo these procedures likely had a better short-term prognosis, while those with the most severe disease may have died before the interventions could be performed. Nevertheless, a true survival benefit, particularly in the amputation group, cannot be ruled out, as removal of ischemic or infected tissue may reduce systemic inflammatory burden and mortality risk. When revascularization fails to achieve tissue salvage, timely amputation should not be delayed (22). Whenever possible, minor amputations are preferred, as more extensive procedures may substantially compromise quality of life.

In the current literature, there is considerable heterogeneity in reported mortality among patients with CLTI, as various treatment modalities are analyzed, including conservative management, surgical revascularization, endovascular therapy, combined revascularization, and post-amputation care.

Among CLTI patients older than 70 years treated with either revascularization or conservative management, amputation-related mortality was 44%, 66%, and 85% at 1, 3, and 5 years, respectively (23). However, there are limited data on the impact of amputation on patient survival following endovascular treatment.

Mortality risk in patients with chronic kidney disease was almost 1.7 times higher, and a positive association between creatinine and mortality was confirmed. Creatinine reflects renal function and nutritional-metabolic reserve. Its persistently elevated levels are associated with chronic kidney disease, which represents proinflammatory and prothrombotic state characterized by increased oxidative stress, elevated levels of matrix metalloproteinases, and the accumulation of uremic toxins, all of which accelerate endothelial damage and atherosclerosis (24, 25). CLTI patients with chronic kidney disease had lower revascularization rates, higher in-hospital mortality, increased risk of vascular complications, bleeding, and amputation compared to those without chronic kidney disease (23, 24).

Creatinine has also been identified as a predictor of mortality after cardiac surgery and percutaneous coronary intervention, and higher creatinine levels have been associated with worse survival outcomes in patients with colorectal, renal, and ovarian cancers, as well as sarcoma (26–28). Conversely, low or borderline creatinine values may reflect sarcopenia and protein-energy malnutrition, associated with chronic inflammation, immobility and catabolism.

Fibrinogen plays a role in the pathogenesis of atherosclerosis and atherosclerotic complications (29). Ischemia, tissue necrosis, and infections lead to chronic cytokine activation which increases fibrinogen. This reflects immuno-inflammatory response and prothrombotic state with impaired microcirculation, increased platelet aggregation, higher blood viscosity, and thrombus resistance to fibrinolysis contributing to distal perfusion failure (29, 30). Patients with PAD and elevated fibrinogen levels have been documented to have a higher risk of ischemic stroke, bleeding, amputation, and mortality (31). A study conducted by Doweik et al. confirmed higher cardiovascular and total mortality in patients with PAD with elevated fibrinogen concentrations (32). Higher fibrinogen is related to worse overall survival in malignant diseases (33). Taken together, fibrinogen and creatinine reflect immuno-inflammatory and nutritional-metabolic status and may represent indicators of higher mortality risk in patients with CLTI.

Long-term outcomes in patients undergoing percutaneous revascularization could be assessed using the Naples Prognostic Score, which reflects the patient's nutritional, immunological status, and systemic inflammatory response, by analyzing serum albumin, total cholesterol, neutrophil to lymphocyte ratio, and lymphocyte to monocyte ratio (34, 35).

While composite scores are designed to determine risk through multidimensional indices, the evaluation of individual biomarkers such as creatinine and fibrinogen allows for more direct interpretation. These routinely available parameters reflect key pathophysiological pathways and may provide prognostic information. However, a major limitation of single biomarkers is their susceptibility to variability during acute diseases and metabolic fluctuations, whereas composite scores tend to demonstrate greater stability.

Our study expands the knowledge of CLTI patients treated endovascularly and may be considered in future meta-analyses. A multidisciplinary approach, including infection management with debridement and minor amputations, treatment of limb ischemia, and optimal management of comorbidities is essential to improve survival outcomes.

However, our study has several limitations that may have affected the results. The major limitation of our study is that it included only patients with ischemic ulcers who underwent EVT; patients treated with open surgery, conservatively, patients without the option of revascularization, and patients undergoing primary amputation were not included, which limits the generalizability of our findings to all patients with CLTI. The data were evaluated retrospectively, and patients were treated at a single center, which may have introduced selection bias. Other limitations include sample size and underrepresentation of women. The number of smokers may have been underestimated; however, many patients were likely passive smokers due to permissive attitudes and lack of restrictive measures in past decades. Causes of death were not assessed because of the low autopsy rate in our population. Patient adherence to follow-up was not evaluated, but given the older age, impaired mobility, presence of ulcers, pain, and polymorbidity, it was likely suboptimal. When analyzing the impact of amputations and reinterventions on mortality, time-dependent covariate analysis was not realized due to the retrospective design, limited temporal resolution and heterogeneous timing of interventions. Therefore the results should be interpreted as hypothesis-generating rather than causal. Another limitation is the inability to account for residual confounding factors, including frailty, low socioeconomic status, and treatment delays. Frail patients are more prone to complications and experience delayed ulcer healing, while individuals with lower socioeconomic status or limited access to healthcare are at increased risk of inadequate or delayed treatment.

## Data Availability

The raw data supporting the conclusions of this article will be made available by the authors, without undue reservation.
